# Longitudinal tracking of human plasma oxytocin suggests complex responses to moral elevation

**DOI:** 10.1016/j.cpnec.2021.100105

**Published:** 2021-12-22

**Authors:** Luke Parkitny, C. Sue Carter, Melissa K. Peckins, Deirdre Ann Hon, Sarina Saturn, H.P. Nazarloo, William Hurlbut, Brian Knutson, Steven Crane, Xiola Harris, Jarred Younger

**Affiliations:** a Department of Psychology, The University of Alabama at Birmingham, Birmingham, AL, 35294, USA; b Departments of Pediatrics, Neurology and Neuroscience, Baylor College of Medicine, Houston, TX, 77030, USA; c Jan and Dan Duncan Neurological Research Institute at Texas Children’s Hospital, Houston, TX, 77030, USA; d Kinsey Institute, Indiana University, Bloomington, IN, 47405, USA; e Department of Psychology, St. John’s University, Queens, NY, 11439, USA; f School of Education, University of Portland, Portland, OR, 97203, USA; g Department of Psychological Sciences, University of Portland, Portland, OR, 97203, USA; h Department of Neurobiology, Stanford University, Stanford, CA, 94305, USA; i Department of Psychology, Stanford University, Stanford, CA, 94305, USA

**Keywords:** Oxytocin, Moral elevation

## Abstract

Positive social experiences may induce oxytocin release. However, previous studies of moral elevation have generally utilized cross-sectional and simple modeling approaches to establish the relationship between oxytocin and emotional stimuli. Utilizing a cohort of 30 non-lactating women (aged 23.6 ± 5.7 years), we tested whether exposure to a video identified as capable of eliciting moral elevation could change plasma oxytocin levels. Uniquely, we utilized a high-frequency longitudinal sampling approach and multilevel growth curve modeling with landmark registration to test physiological responses. The moral elevation stimulus, versus a control video, elicited significantly greater reports of being “touched/inspired” and “happy/joyful”. However, the measured plasma oxytocin response was found to be markedly heterogeneous. While the moral elevation stimulus elicited increased plasma oxytocin as expected, this increase was only modestly larger than that seen following the control video. This increase was also only present in some individuals. We found no relationship between plasma oxytocin and self-report responses to the stimulus. From these data, we argue that future studies of the relationship between oxytocin and emotion need to anticipate heterogeneous responses and thus incorporate comprehensive individual psychological data; these should include evidence-based variables known to be associated with oxytocin such as a history of trauma, and the individual’s psychological and emotional state at the time of testing. Given the complexity of physiological oxytocin release, such studies also need to incorporate frequent biological sampling to properly examine the dynamics of hormonal release and response.

## Highlights

• A moral elevation stimulus elicits positive emotional responses
• The human oxytocin responses to a morel elevation stimulus are more heterogenous than previously reported
• Future studies need to utilize longitudinal hormone measurements and comprehensive psychological assessments

## 1 Introduction

Oxytocin is a versatile neuropeptide that regulates diverse aspects of human physiology and behavior, including parturition, lactation, pain, sexual, and social behaviors [[1], [2], [3]]. Much attention has been directed towards characterizing how oxytocin moderates positive social and emotional functions such as trust, compassion, and empathy [4,5]. However, seemingly contrary social effects of oxytocin have also been reported and include attenuation of trust [6], increased threat perception [7], and deviation from fairness norms [8]. These complex interactions between oxytocin and behavior appear to be driven by factors such as environmental and social context, the dosage of the exogenously administered hormone, time course, the individual’s emotional history, and whether the peptide is naturally released or exogenously administered [[9], [10], [11], [12]]. The effects of oxytocin also appear to be sexually dimorphic, mediated by receptor variability, interactions with the associated neuropeptide vasopressin [3,13], and affected by life events such as maltreatment and trauma [10,12,14].

Examinations of the relationship between oxytocin and behavior traditionally utilize one of two experimental paradigms. In the first approach, the intranasally-administered exogenous hormone is delivered and subsequent behavioral responses are quantified. The main limitations of this approach are that it necessitates the use of exogenous hormone and that the dosing characteristics probably do not completely parallel physiological hormone release. In the second approach, endogenous oxytocin levels in the blood are measured in response to specific stimuli that are selected to elicit emotional responses, such as social vocalization [15], empathy [16], or warm partner contact [17]. Specific and reliable stimuli of oxytocin release have proven difficult to identify, although several studies do link sexual stimulation and intense physical or emotional stressors to the release of oxytocin [9,18,19]. Another source of difficulty arises from the natural human variability in response to psychological and emotional stimuli, possibly due to differences in the history of individuals and genetic variability [20].

In addition, it has been demonstrated that oxytocin release is a complex event that is best modeled by frequently sampled longitudinal data. Animal data suggest that oxytocin is released in a pulsatile manner and that its half-life in blood is around 1–5 min. Studies applying cross-sectional and similarly limited frequency sampling approaches are thus unlikely to adequately model rapid physiological responses to specific stimuli [21]. More frequent sampling has tended to show idiosyncratic patterns of oxytocin release. For example, measurements taken at 5-min intervals in response to music or the cold pressor test have revealed marked pulsatile increases in oxytocin in some, but not all, participants [22]. Unfortunately, with few exceptions, studies of stimulus-induced endogenous oxytocin release have utilized limited sampling, typically taken at arbitrary intervals of 15–60 min following stimulus presentation. Finally, it is important to recognize that while oxytocin exerts its behavioral effects by targeting the brain, it is most usually measured in plasma from venous blood. Yet the relationship between central and systemic oxytocin is rarely defined.

Thus, a well-defined stimulus, frequent sampling, and careful characterization of the emotional history of participants are necessary to detect stimulus-associated changes. As such, in this paper, we present the results of a study in which we attempted to model the endogenous systemic oxytocin response to an experimental positive emotional stimulus. Based on existing published data [23,24], we expected that an uplifting audiovisual stimulus would result in a rapid and unambiguous oxytocin elevation in the blood; we hoped to exploit this response to measure its subsequent effect on behavior. However, due to complex and markedly heterogeneous oxytocin response patterns, we instead focused our attention on modeling oxytocin levels and identifying responders to a morally uplifting stimulus video.

## 2 Material and methods

### 2.1 Participants

Study procedures were conducted according to a protocol approved by the institutional review board at the University of Alabama at Birmingham (UAB). Eligible consenting participants were healthy females, 18–39 years of age, from the Birmingham metropolitan area in Alabama, USA. Exclusion criteria included current pregnancy or breastfeeding, any diagnosed psychological or psychiatric disorder, any illness within 30 days of participation, a history of substance abuse, regular use of prescribed medication, or any acute pain or history of chronic pain.

### 2.2 Data collection procedures

Study eligibility was determined through a telephone interview. Eligible individuals attended the UAB Clinical Research Unit (CRU) for two sessions, held approximately one week apart. In each session, individuals were presented with either a moral-elevation video or an emotionally neutral control video (see Supplementary File). The video presentation order was randomized.

**Fig. 1:**
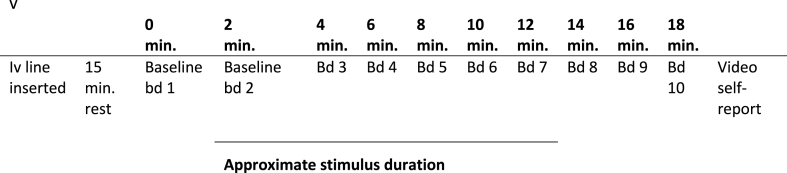
Time-line of each experimental session. The two video stimuli commenced upon the successful collection of the second baseline draw and were 9 min. 45 sec. duration for the control session, and 9 min. 12 sec. for the moral elevation session. Between the insertion of the intravenous line (iv) and the completion of the self-report, individuals remained in complete silence. Blood draws (bd) were obtained every 2 min according to the described protocol.

The sessions were designed to minimize interpersonal communication and other potential sources of interpersonal stimuli. All sessions were conducted in a quiet, private clinical room in the presence of one research nurse and one male investigator. Participants were seated in an armchair in front of a 17-inch computer monitor with one arm extended on the armrest. An intravenous cannula was inserted into a vein in the antecubital fossa for blood collection. The participant was instructed to not communicate with research staff until the session was completed unless they experienced physical discomfort. As much as practicable, the research staff remained out of sight of the participant throughout the session. Circumaural headphones were used to suppress ambient noise and increase soundtrack clarity. After the intravenous cannula was sited, participants were asked to focus on the monitor; all subsequent instructions were provided via remotely controlled text displayed on the screen. Participants initially rested in silence during a 15-min habituation period. Following this, the blood draws were commenced and repeated every 2 min for a total of ten 8 ml draws over 18 min. There were two baseline draws, six during the stimulus video, and two post-stimulus (Fig. 1). After the video, participants continued to rest quietly until all blood draws were completed. The participant then completed a written post-video questionnaire in which they evaluated their emotional response to the stimulus. Except for the collection of personal and demographics data (only done in Session 1), the procedures were identical in both sessions.

### 2.3 Experimental stimuli

The moral elevation and control videos were intended to elicit feelings of moral elevation. These videos were selected based on the results of prior published studies and an online pilot study conducted before the commencement of the main study. For this project, we defined moral elevation as a specific emotional state elicited by witnessing compassionate acts in others [25,26]. As such, the moral elevation videos shared the common theme of people performing and responding to altruistic acts.

The moral elevation videos (MEV) included a segment from the Oprah Winfrey show, in which teachers are lauded by former students for their mentorship; this video has been used in previous studies to induce moral elevation [24,27,28]. The second video featured a Thai Life Insurance advertisement *Unsung Hero* (2014) in which a young man performs a series of selfless acts and is ultimately made aware of the positive effects of his actions (https://www.youtube.com/watch?v=uaWA2GbcnJU). The control video (COV) included three demonstrations of do-it-yourself construction obtained from YouTube: a battery being constructed from common household items, woodwork building of a stool-style chair, and the steps to fold an origami crane. The MEV and COV presentations were approximately time-matched to allow us to make comparisons of temporal effects.

### 2.4 Self-report measures and donation task

Basic demographic and socioeconomic data were collected using a standardized form. Emotional responses to the stimuli were evaluated based on answers to 11 items on a 7-point scale anchored at *“not at all”* and *“extremely”* [29]. The items included the following: “how pleasant was the video?,” “how interesting was the video?,” “how emotionally affecting was the video?,” and the extent to which the participant felt “touched/inspired,” “amused,” “happy/joyful,” “calm/content,” “sad,” “angry,” “disgusted,” and “afraid.” The *a priori* selected primary determinants of self-reported moral elevation were “touched/inspired” and “happy/joyful”.

We also included an unprompted donation task to quantify behavioral responses. This paralleled earlier work that showed that exogenously administered oxytocin may increase donation, albeit with moderation by personal background factors [30]. After each experimental session, the participant was provided their study compensation, part of which was in $1 notes. A clear donation box for St. Jude’s Children’s Hospital showing an image of a young child with cancer (permission obtained from the hospital) was located within 1 m of the seating area. The box was loaded with several notes beforehand (serial numbers recorded). The individual was then left alone for 2 min while the investigator performed administrative tasks out of sight. Upon the participant’s departure, the money was retrieved and the donation amount was determined based on a comparison of note/coin quantity and note serial numbers. The participant was not informed that the donation task was a part of the study until a debriefing at the end of the study.

### 2.5 Oxytocin sampling and quantification

To minimize proteolytic degradation of oxytocin, K_2_EDTA blood tubes were pre-chilled overnight and then, during the experimental session, kept on wet ice except when the blood was being drawn. Saline was used to flush the cannula after each draw and a waste tube was drawn just before obtaining each experimental sample to avoid sample dilution and contamination. Immediately upon completion of the session, all collected tubes were centrifuged at 4°C, aliquoted into pre-chilled cryotubes, then transferred to a −80°C freezer until analysis (approximately one year maximum).

Upon study completion, plasma samples were shipped overnight on dry ice to Indiana University for analysis. Plasma oxytocin quantification was performed using Enzo Life Sciences ELISA kits. All samples were run in duplicate and the mean concentrations were used in the analysis. The enzyme immunoassay (EIA) procedures have been previously validated for parallelism, spike-recovery, and cross-reactivity/specificity [31]. The EIA protocol without oxytocin extraction had been criticized since it provides estimates that are orders of magnitude greater than methods employing extraction [32]. However, new evidence suggests that this discrepancy in estimates can result in part from the removal of bound oxytocin during the extraction step [[33], [34], [35]]. Oxytocin has a high affinity for binding to blood plasma proteins [37], and procedures that break these bonds before measurement provide estimates of plasma oxytocin consistent with levels found in unextracted samples: 500–1200 pg/ml [36,37]. Further, it has been shown that solid-phase extraction artificially reduces oxytocin levels in plasma samples [38,39], and high-performance liquid chromatography has illustrated that the amount of authentic peptide labeled as oxytocin in these extracted samples can be as low as 7% [32], the rest comprising cross-reactive molecules which are yet to be identified. As reviewed in MacLean et al. [35,40], critiques of the “lack of validity” of assays using unextracted plasma samples [41] may have been premature. For example, two recent studies [42,43] compared the usefulness in predicting human behavior of extracted versus unextracted plasma. It is important to note that one of those studies describing the necessity for extraction [41], was conducted in the laboratory of the author an earlier critique [43]. Both of these recent studies revealed, in direct comparisons, that assays of unextracted samples were more likely than extracted samples to show associations with behavior. The source of this apparent failure to confirm the criticism of McCullough et al. [41] is not known. However, it is possible that after extraction the levels of peptide remaining are sometimes not sufficient to allow a reliable measure of oxytocin. Furthermore, our extensive experience with the measurement of oxytocin in unextracted plasma indicates that reliable and replicable relationships exist between peripheral measures of oxytocin and behavior [44,45], even in analyses done several years apart [46]. This evidence lends support to the idea that enzyme immunoassay without extraction reflects a useful assessment of oxytocin in human blood plasma that can be associated with behavior. For these and other reasons detailed in [35], we opted in the present study to not extract oxytocin prior to quantification assays.

### 2.6 Statistical analyses

Expecting the stimulus condition to increase the levels of plasma oxytocin, we initially tested for differences between plasma oxytocin between the two stimulus conditions by using linear mixed models (LMM) with an appropriate covariance structure selected based on a low Akaike’s Information Criterion. These models appropriately test for repeated-measures longitudinal data effects. Further examinations of plasma oxytocin peaks were carried out by plotting the data obtained from the stimulus and control video sessions and visual observation. Models were generated using SPSS v23 and significance was determined using a p-value threshold of 0.05.

Due to the complex and ambiguous results found in the initial analyses, we then employed an analytic strategy that was more sensitive to individual differences. We modeled the plasma oxytocin response to the control and moral elevation videos using two-piece multilevel growth curve modeling with landmark registration (GCM-LR), a method that has been previously applied to neuroendocrine data to study salivary cortisol and salivary alpha-amylase responses to a stressor [47,48]. The benefit of using two-piece multilevel GCM-LR to study the oxytocin response to stimuli is that it accounts for interindividual variability in the time that it takes participants to reach their peak oxytocin response and allows us to examine the reactivity, peak, and recovery phases of the oxytocin response separately. We identified plasma oxytocin peaks by plotting the data obtained from the COV and MEV sessions and selecting the first peak that was followed by a decline or plateau [47]. If the first peak was followed by a plateau, we selected the sample within the plateau that represented at least a 10% increase from the first identified peak. Participants were labeled a “responder” if their peak sample represented at least a 15% increase in oxytocin from baseline levels. Our decision to use a 15% threshold to identify responders was based on recent work showing that on average, salivary oxytocin levels increased by 15% in response to an emotional video [49]. Participants were labeled a “non-responder” if their plasma oxytocin did not increase by at least 15% above baseline levels throughout the session. For responders, we created a ‘peak time’ variable that was set to responders’ peak sample time. For non-responders, ‘peak time’ was set to the mode peak sample time of responders (control video = 4 min, moral elevation video = 8 min; [47]). Oxytocin data were winsorized to 3SD from the mean (*n* = 2) and log-transformed.

Two-piece multilevel GCM-LR models were fit with SAS® software (Version 9.4) using PROC MIXED [50] with restricted maximum likelihood estimation and an unstructured covariance structure. An unstructured covariance structure was used because the models did not converge with a first-order autoregressive covariance structure. We included samples 2–10 (2–18 min) in growth models and sample 1 was included as a covariate to control for baseline oxytocin levels. First, for each experimental condition, we fit unconditional models with the intercept set to each individual’s peak time (non-responders set to the mode peak time of responders) and included baseline oxytocin (0 min), minutes to peak (oxytocin reactivity slope), and minutes after the peak (oxytocin recovery slope) with random intercepts and slopes [47]. Next, we tested whether video order (0 = first, 1 = second video) was associated with the phases of oxytocin response, including peak activation, reactivity slope, and recovery slope. Peak activation represents the level of oxytocin at the apex of the response curve. Reactivity and recovery slopes represent the slope of oxytocin toward and away from participants’ peak activation, respectively [47]. Finally, following the approach used by Almeida, et al. [51], we created a video variable (0 = COV, 1 = MEV) and included it as a covariate in our model to test for differences in the oxytocin response between the two experimental conditions with and without controlling for video order.

## 3 Results

### 3.1 Participants

The study cohort included 30 women aged 23.6 ± 5.7 years old. Of these, 13 identified as African-American, 12 as Caucasian, five as Asian, and one individual endorsed “other.” Ten additional women did not complete the protocol; eight individuals were lost because we were unable to secure adequate intravenous access and two additional individuals exhibited vasovagal syncope associated with venipuncture and were withdrawn from the study by the investigator.

### 3.2 Self-report response and donation task

The MEV stimulus elicited significantly greater reports of being “touched/inspired” (6.07 ± 1.4 vs. 2.45 ± 1.8, p < 0.001) and “happy/joyful” (5.5 ± 1.5 vs. 2.5 ± 1.8, p < 0.001) versus COV, thus validating the stimulus conditions. We did not analyze the donation task as only 2 individuals chose to donate.

### 3.3 Plasma oxytocin

The immunoassay coefficients of variation were 7.07% (intra-assay) and 2.35% (inter-assay). In our primary analyses, differences in plasma oxytocin levels between the emotional and control videos were tested using an LMM with a first-order autoregressive covariance structure. The differences between the MEV and COV sessions were not significant (F = 3.73, p = 0.055). To illustrate the time-course of plasma oxytocin, in the Supplementary File we have provided plots of the group-average and individual oxytocin time-course data.

In our secondary analyses, using an oxytocin increase above the baseline of ≥15% as a cut-off, we classified 12 participants (40%) as responders and 18 participants (60%) as non-responders. The average increase in oxytocin from baseline to peak for responders to the COV was 30.9% above baseline levels. For the MEV, eight participants (26.7%) were classified as responders and 22 participants (73.33%) were classified as non-responders. The average percent increase in oxytocin from baseline to peak for responders to the MEV was 41.4%. Four participants (13.3%) were classified as responders for both COV and MEV, 14 participants (46.67%) were classified as non-responders for both COV and MEV, and 12 participants (40%) switched responder status between videos (*n* = 8 were classified as responders to the COV and non-responders to the MEV; *n* = 4 were classified as non-responders to the COV and responders to the MEV). Responder status was not associated with baseline oxytocin levels during the COV (0 min, t = 1.89, p = 0.069; 2 min, t = 1.15, p = 0.26); however, responders to the MEV had significantly lower oxytocin levels at 0 min (t = 3.03, p = 0.005) and at 2 min (t = 2.13, p = 0.042) compared to non-responders. For both videos, responder status was not associated with participants’ ratings of how emotionally affecting the video was (COV t = 0.22, p = 0.83; MEV t = 0.08, p = 0.94) or how touched/inspired the video made them feel (COV t = 0.91, p = 0.37; MEV t = 1.37, p = 0.18). Individual plots for plasma oxytocin for each session are provided in the Supplementary File.

#### 3.3.1 Oxytocin response to the COV

Only peak activation in response to the COV was significant, *b* = 7.15, SE = 0.03, *p* < 0.001 (Table 1). The change in plasma oxytocin from baseline to peak, *b* = 0.001, SE = 0.01, *p* = 0.88 and post-peak decline, *b* = −0.002, SE = 0.002, *p* > 0.32 were not significant. Order was not associated with change in oxytocin in response to the COV (*p*s = .43 to .51). Order was not associated with peak activation, (*b* = 0.10, SE = 0.05, *p* = 0.06), although participants who viewed the MEV second, trended toward increased oxytocin levels at peak activation compared to participants who viewed the MEV video first.

**Table 1:** Estimates for growth curve models of the oxytocin response to control and moral elevation videos.

	Control Video				Moral Elevation Video				Control vs. Moral Elevation Video			
	Baseline Only Model		Order Model		Baseline Only Model		Order Model		Video Condition Model		Order Model	
	b	SE	b	SE	b	SE	b	SE	b	SE	b	SE
Intercept	7.15**	0.03	7.09**	0.04	7.13**	0.03	7.09**	0.03	7.15**	0.02	7.11**	0.03
Baseline Oxytocin	0.44**	0.14	0.14	0.14	0.55**	0.08	0.35*	0.16	0.27**	0.08	0.21*	0.09
Time Before Peak	0.001	0.01	−0.004	0.01	−0.004	0.01	−0.01^Ŧ^	0.01	0.01	0.01	−0.001	0.01
Time After Peak	−0.002	0.002	−0.004	0.003	0.002	0.003	0.01	0.004	−0.003	0.002	−0.002	0.002
Order	–	–	0.10 ^Ŧ^	0.05	–	–	0.08*	0.03	–	–	0.08**	0.02
Baseline Oxytocin × Order	–	–	0.33	0.24	–	–	0.19	0.20	–	–	0.14	0.10
Time Before Peak × Order	–	–	0.01	0.02	–	–	0.01	0.01	–	–	0.01 ^Ŧ^	0.01
Time After Peak × Order	–	–	0.003	0.01	–	–	−0.01	0.01	–	–	−2.00 × 10^−5^	0.003
Video	–	–	–	–	–	–	–	–	−0.02	0.02	−0.02	0.02
Baseline Oxytocin × Video Condition	–	–	–	–	–	–	–	–	−0.26**	0.09	0.03	0.10
Time Before Peak × Video Condition	–	–	–	–	–	–	–	–	−0.01 ^Ŧ^	0.01	−0.01	0.01
Time After Peak × Video Condition	–	–	–	–	–	–	–	–	0.01 ^Ŧ^	0.003	0.005 ^Ŧ^	0.003

Note. ^Ŧ^ p < 0.10, *p < 0.05, **p < 0.01. Baseline oxytocin was collected at 0 min. The first video (order) and Control Video (video condition) were the reference categories.

#### 3.3.2 Oxytocin response to the MEV

Only peak activation in response to the MEV was significant, *b* = 7.13, SE = 0.03, *p* < 0.001. There was no significant change in plasma oxytocin from baseline to peak, *b* = −0.004, SE = 0.01, *p* > 0.50, and post-peak decline, *b* = 0.002, SE = 0.003, *p* > 0.52. Order was only associated with peak activation, *b* = 0.08, SE = 0.03, *p* = 0.02. Participants who viewed the MEV second had increased oxytocin levels at peak activation compared to participants who viewed the MEV first.

#### 3.3.3 COV vs. MEV

The video condition was not significantly associated with the oxytocin response; however, findings were trending toward significance (Table 1). There was a less steep increase in oxytocin from baseline to peak, *b* = −0.01, SE = 0.01, *p* = 0.07, and less steep post-peak decline in oxytocin, *b* = 0.01, SE = 0.003, *p* = 0.05, during the MEV compared to the COV. The video condition was not associated with peak activation, *b* = −0.02, SE = 0.02, *p* = 0.23. When order was added to the model, there was only a marginally significant finding between video condition and post-peak decline in oxytocin, *b* = 0.005, SE = 0.003, *p* = 0.09. Video condition was not associated with a change in oxytocin from baseline to peak and peak activation when controlling for order (*p* values of 0.16–0.31).

## 4 Discussion

This study was designed to characterize the time-course and test the homogeneity of plasma oxytocin responses to an audiovisual stimulus that had been previously defined as eliciting a sense of moral elevation. Our primary aim was to test modeling approaches that will benefit future studies aiming to capture the temporal characteristics of hormonal responses to social stimuli. In short, we found that while the MEV stimulus elicited increased plasma oxytocin, this was only modestly larger than the increase seen following the COV and was present in a smaller number of individuals. In addition, our self-reported behavioral measures of emotional reactivity did not predict the patterns of hormonal outcomes.

Moral elevation induced by the presentation of videos similar to that used in the present study has been reported to induce milk ejection and nursing behavior and was thus expected to promote oxytocin release [24]. However, studies of nursing women did not measure plasma oxytocin and it is not known how well the nervous systems of lactating women translate to general physiology; for instance, hormonal priming for milk ejection induces higher sensitivity to emotionally evocative experiences in lactating compared to non-lactating women [21,52]. Earlier studies with both typical participants [53], those with a history of maltreatment [10,20], and the genetic disorder known as Williams Syndrome [22] have also shown distinct patterns of individual variation in oxytocin that may be subject to various physiological modifications.

Despite the lack of a homogeneous response, we were able to describe the oxytocin time-course among those individuals showing a task-related oxytocin increase. There were more responders to the COV (n = 12, 40%) than the MEV (n = 8, 26.67%). However, the mean percentage increase in oxytocin was greater in responders to the MEV (41.43%) than responders to the COV (30.86%). To further illustrate the marked homogeneity in responses, we have included plots of plasma oxytocin for each individual in the Supplementary File. Firstly, we detect differences in the timing of the expected oxytocin spike in those individuals who appear to respond to the stimulus condition. For instance, individuals OXT7 and OXT8 display increased oxytocin at ∼12–15 min while individuals OXT14, OXT18, OXT28 show a much earlier response. Secondly, many individuals show no marked change in oxytocin (e.g. OXT5, OXT6) while others show no discernible difference between the two conditions (e.g. OXT9, OXT14). Other individuals show other complex kinetics, again suggesting that individual factors that were not captured in this study are critical in understanding these release characteristics. While we cannot completely resolve the implications of these results with the present data, they offer several important methodological and biological warning signs for research.

Firstly, our study was designed to maximize the emotional effect of the moral elevation stimulus while minimizing undesirable extraneous variables. The video stimuli used here were selected primarily because they have been used in other studies [24,27,28]. In addition, their emotional effects were validated in our pilot study through participant self-report. However, the stimuli may not have been sufficiently emotionally powerful to elicit reliable physiological responses in at least some individuals. To maximize the effects of the MEV, we utilized stringent environmental controls. However, we still found substantial increases in plasma oxytocin following the control condition which may stem from participants’ perceiving the research environment as caring or stressful. Given that not all individuals responded in each condition, responder status may have been driven by variables that were not measured in this study.

Secondly, while most oxytocin studies utilize cross-sectional or limited longitudinal designs, we believe that our data suggest that these approaches increase the risk of false-positive and false-negative findings. We have demonstrated marked individual variability in response and a relatively modest effect of a well-established stimulus protocol. We believe that given a different sample and serendipitously identified baseline and post-stimulus time-points, spurious results may have emerged. The heterogeneity in our participants’ oxytocin response to the stimuli prompted us to use two-piece multilevel growth curve modeling with landmark registration [47]. This statistical approach accounts for individual variability in the time-course of the oxytocin response by aligning participants according to the peak of their response. Thus, we were able to confirm that differences in each phase of the oxytocin response to the moral elevation and control videos were not masked by individual differences in peak latency. Although we did not find a strong or even moderate statistical effect for the moral elevation and control videos with landmark registration, our study highlights the importance of collecting enough post-stimulus time-points to test for such an effect.

Thirdly, our initial aims and study design did not incorporate comprehensive measures of behavioral or psychological variables and/or histories of adversity. However, even in our small cohort of young adult women (n = 30), we observed individual variation in the oxytocin response. This outcome is consistent with earlier reports that plasma oxytocin levels were not changed by an emotional stimulus alone, but rather required a complex interplay between stimulus and other behaviors [5,20]. While we cannot explain what factors determined responder status in this cohort, we suggest that future studies consider collecting comprehensive socioeconomic, emotional history, and physiological variables as part of their study design.

Lastly, there are important unresolved physiological factors that may underlie these complex responses. Most critically, plasma oxytocin levels may not necessarily reflect availability or uptake by relevant receptors in the brain [54]. Studies that have compared oxytocin in cerebrospinal fluid and blood were inconclusive: central and peripheral oxytocin may [55] or may not be correlated [56]. However, these studies have not used the rapid and repeated sampling procedures described here. To properly test these relationships, longitudinal sampling and appropriate timing are critical if, for example, plasma and central oxytocin levels correlate but not in a contemporaneous manner. Serial oxytocin sampling in the cerebrospinal fluid (CSF) is impractical. Some evidence, however, suggests that measurement of oxytocin in saliva may actually be a more reliable reflection of release patterns, and thus more useful as biomarkers for acute behavioral responses [19,31,54,57,58]. In the present study, we detected rapid and transient changes, a fact that needs to be considered in attempts to measure acute changes in blood levels of oxytocin. It is possible that previous studies that have not detected group differences, following stimuli such as massage [59], may have failed to obtain samples during the relatively brief period when oxytocin is released.

## 5 Conclusions

This study suggests that a classic audiovisual moral elevation task is associated with increased plasma oxytocin, but that this effect is modest and heterogeneous. In addition, responses to emotional stimuli, such as those used here, are typically limited in duration. This observation may be useful in designing future research aimed at evaluating the behavioral or physiological consequences of oxytocin. However, heterogeneous behavioral and physiological responses to emotional stimuli are common. As shown here, many individuals fail to show a response after exposure to presumably emotionally evocative experiences. It is not known if these are predominantly state or trait responses or driven by individual differences that could alter reactivity to a stimulus. Exploring the sources of these individual differences will be important for future research into the causes or consequences of the release of oxytocin.

## Author contributions

J. Younger developed the study concept. L. Parkitny, S. Carter, W. Hurlbut, S. Saturn, B. Knutson, S. Crane, J. Younger contributed to the study design. Testing and data collection were performed by L. Parkitny, X. Harris, and J. Younger. L. Parkitny, M. Peckins, and J. Younger performed the data analysis. All authors were involved in interpreting the data. L. Parkitny drafted the manuscript, and all authors provided critical revisions. All authors approved the final version of the manuscript for submission.

## Funding

This work was supported by a grant from the 10.13039/100001614Fetzer Institute, *Moral Elevation, Oxytocin, and Pain.* Assays for this study were supported in part by 10.13039/100000002NIH (P01 HD 075750 to CSC). We also are grateful to Dr. Leila Partoo, Indiana University, for her assistance with the oxytocin assays.

## Conflict of interest

The authors declare no conflicts of interest.

## Declarations of interest

None.

## References

[1] Goodin B.R., Ness T.J., Robbins M.T. (2015). Oxytocin – a multifunctional analgesic for chronic deep tissue pain. Curr. Pharmaceut. Des..

[2] Carter C.S. (2014). Oxytocin pathways and the evolution of human behavior. Annu. Rev. Psychol..

[3] Carter C.S. (2020). Is oxytocin “nature’s medicine”?. Pharmacol. Rev..

[4] MacDonald K., MacDonald T.M. (2010). The peptide that binds: a systematic review of oxytocin and its prosocial effects in humans. Harv. Rev. Psychiatr..

[5] Barraza J.A., Zak P.J. (2009). Empathy toward strangers triggers oxytocin release and subsequent generosity. Ann. N. Y. Acad. Sci..

[6] Ide J.S. (Jul 1 2018). Oxytocin attenuates trust as a subset of more general reinforcement learning, with altered reward circuit functional connectivity in males. Neuroimage.

[7] Leppanen J., Ng K.W., Kim Y.R., Tchanturia K., Treasure J. (Jan 1 2018). Meta-analytic review of the effects of a single dose of intranasal oxytocin on threat processing in humans. J. Affect. Disord..

[8] Radke S., de Bruijn E.R. (2012). The other side of the coin: oxytocin decreases the adherence to fairness norms. Front. Hum. Neurosci..

[9] Hurlemann R., Grinevich V. (2018).

[10] Pierrehumbert B., Torrisi R., Laufer D., Halfon O., Ansermet F., Popovic M.B. (2010). Oxytocin response to an experimental psychosocial challenge in adults exposed to traumatic experiences during childhood or adolescence. Neuroscience.

[11] Bartz J.A., Zaki J., Bolger N., Ochsner K.N. (2011). Social effects of oxytocin in humans: context and person matter. Trends Cognit. Sci..

[12] Bakermans-Kranenburg M.J., van IJzendoorn M.H., Riem M.M., Tops M., Alink L.R. (2011). Oxytocin decreases handgrip force in reaction to infant crying in females without harsh parenting experiences. Soc. Cognit. Affect Neurosci..

[13] Herbert Z., Sauer B., Jirikowski G. (2019). Sexual dimorphism in rat oxytocinergic hypothalamic regions. Eur. J. Anat..

[14] Ellis B.J., Horn A.J., Carter C.S., van Ijzendoorn M.H., Bakermans-Kranenburg M.J. (2021/06/01/2021). Developmental programming of oxytocin through variation in early-life stress: four meta-analyses and a theoretical reinterpretation. Clin. Psychol. Rev..

[15] Seltzer L.J., Ziegler T.E., Pollak S.D. (2010). Social vocalizations can release oxytocin in humans. Proc. R. Soc. Lond. B Biol. Sci..

[16] Barraza J.A., Zak P.J. (Jun 2009). Empathy toward strangers triggers oxytocin release and subsequent generosity. Ann. N. Y. Acad. Sci..

[17] Grewen K.M., Girdler S.S., Amico J., Light K.C. (2005). Effects of partner support on resting oxytocin, cortisol, norepinephrine, and blood pressure before and after warm partner contact. Psychosom. Med..

[18] C. S. Carter, "Oxytocin and sexual behavior," (in eng), Neurosci. Biobehav. Rev.*,* vol. 16, no. 2, pp. 131-144, Summer 1992. 10.1016/s0149-7634(05)80176-9 1630727

[19] Jong T.R. (Dec 2015). Salivary oxytocin concentrations in response to running, sexual self-stimulation, breastfeeding and the TSST: the Regensburg Oxytocin Challenge (ROC) study. Psychoneuroendocrinology.

[20] Riem M.M.E., De Carli P., van I.M.H., Linting M., Grewen K.M., Bakermans-Kranenburg M.J. (Nov 2017). Emotional maltreatment is associated with atypical responding to stimulation of endogenous oxytocin release through mechanically-delivered massage in males. Psychoneuroendocrinology.

[21] Augustine R.A., Seymour A.J., Campbell R.E., Grattan D.R., Brown C.H. (2018). Integrative neuro‐humoral regulation of oxytocin neuron activity in pregnancy and lactation. J. Neuroendocrinol..

[22] Dai L., Carter C.S., Ying J., Bellugi U., Pournajafi-Nazarloo H., Korenberg J.R. (2012). Oxytocin and vasopressin are dysregulated in Williams Syndrome, a genetic disorder affecting social behavior. PLoS One.

[23] Barraza J., Zak P. (2009). Empathy toward strangers triggers oxytocin release and subsequent generosity. Ann. N. Y. Acad. Sci..

[24] Silvers J.A., Haidt J. (2008). Moral elevation can induce nursing. Emotion.

[25] Haidt J. (2000).

[26] Saturn S.R. (2017). Two factors that fuel compassion: the oxytocin system and the social experience of moral elevation. Oxf. Handb. Compassion Sci..

[27] Schnall S., Roper J., Fessler D.M. (2010). Elevation leads to altruistic behavior. Psychol. Sci..

[28] Piper W.T., Saslow L.R., Saturn S.R. (2015). Autonomic and prefrontal events during moral elevation. Biol. Psychol..

[29] Piper W.T., Saslow L.R., Saturn S.R. (May 2015). Autonomic and prefrontal events during moral elevation. Biol. Psychol..

[30] van Ijzendoorn M.H., Huffmeijer R., Alink L.R.A., Bakermans-Kranenburg M.J., Tops M. (2011). The impact of oxytocin administration on charitable donating is moderated by experiences of parental love-withdrawal. Front. Psychol..

[31] Carter C. (2007). Behavioral associations and potential as a salivary biomarker. Ann. NY Acad. Sci..

[32] Szeto A. (2011). Evaluation of enzyme immunoassay and radioimmunoassay methods for the measurement of plasma oxytocin. Psychosom. Med..

[33] Vehus T. (2016). Versatile, sensitive liquid chromatography mass spectrometry–Implementation of 10 μm OT columns suitable for small molecules, peptides and proteins. Sci. Rep..

[34] Brandtzaeg O.K. (2016). Proteomics tools reveal startlingly high amounts of oxytocin in plasma and serum. Sci. Rep..

[35] MacLean E.L., Wilson S.R., Martin W.L., Davis J.M., Nazarloo H.P., Carter C.S., eng (2019). Challenges for measuring oxytocin: the blind men and the elephant?. Psychoneuroendocrinology.

[36] Whittington K., Connors B., King K., Assinder S., Hogarth K., Nicholson H. (2007). The effect of oxytocin on cell proliferation in the human prostate is modulated by gonadal steroids: implications for benign prostatic hyperplasia and carcinoma of the prostate. Prostate.

[37] Cherepanov S.M. (2021). An improved sample extraction method reveals that plasma receptor for advanced glycation end-products (RAGE) modulates circulating free oxytocin in mice. Peptides.

[38] Furman D.J., Chen M.C., Gotlib I.H. (2011). Variant in oxytocin receptor gene is associated with amygdala volume. Psychoneuroendocrinology.

[39] Kirsch P. (2005). Oxytocin modulates neural circuitry for social cognition and fear in humans. J. Neurosci..

[40] Brandtzaeg O.K. (2016/08/16 2016). Proteomics tools reveal startlingly high amounts of oxytocin in plasma and serum. Sci. Rep..

[41] McCullough M.E., Churchland P.S., Mendez A.J. (Sep 2013). Problems with measuring peripheral oxytocin: can the data on oxytocin and human behavior be trusted?. Neurosci. Biobehav. Rev..

[42] Chu C., Hammock E.A.D., Joiner T.E. (2020). Unextracted plasma oxytocin levels decrease following in-laboratory social exclusion in young adults with a suicide attempt history. J. Psychiatr. Res..

[43] Saxbe D., Khaled M., Horton K.T., Mendez A.J. (Oct 2019). Maternal prenatal plasma oxytocin is positively associated with prenatal psychological symptoms, but method of immunoassay extraction may affect results. Biol. Psychol..

[44] Ebner N.C. (Feb 2019). Associations between oxytocin receptor gene (OXTR) methylation, plasma oxytocin, and attachment across adulthood. Int. J. Psychophysiol..

[45] Roels R., Rehman U.S., Carter C.S., Nazarloo H.P., Janssen E. (May 14 2021). The link between oxytocin plasma levels and observed communication behaviors during sexual and nonsexual couple discussions: an exploratory study. Psychoneuroendocrinology.

[46] Lancaster K. (2015). Plasma oxytocin explains individual differences in neural substrates of social perception. Front. Human Neurosci. Original Res..

[47] Lopez-Duran N.L., Mayer S.E., Abelson J.L. (2014). Modeling neuroendocrine stress reactivity in salivary cortisol: adjusting for peak latency variability. Stress.

[48] Katz D.A., Peckins M.K. (2017). Cortisol and salivary alpha-amylase trajectories following a group social-evaluative stressor with adolescents. Psychoneuroendocrinology.

[49] Procyshyn T.L., Watson N.V., Crespi B.J. (2020). Experimental empathy induction promotes oxytocin increases and testosterone decreases. Horm. Behav..

[50] Singer J.D. (1998). Using SAS PROC MIXED to fit multilevel models, hierarchical models, and individual growth models. J. Educ. Behav. Stat..

[51] Almeida D.M., Lee S., Walter K.N., Lawson K.M., Kelly E.L., Buxton O.M. (2018). The effects of a workplace intervention on employees' cortisol awakening response. Community Work. Fam..

[52] Feldman R., Weller A., Zagoory-Sharon O., Levine A. (2007). Evidence for a neuroendocrinological foundation of human affiliation: plasma oxytocin levels across pregnancy and the postpartum period predict mother-infant bonding. Psychol. Sci..

[53] Gouin J.-P. (2010). Marital behavior, oxytocin, vasopressin, and wound healing. Psychoneuroendocrinology.

[54] Jurek B., Neumann I.D. (2018). The oxytocin receptor: from intracellular signaling to behavior. Physiol. Rev..

[55] Carson D. (2015). Cerebrospinal fluid and plasma oxytocin concentrations are positively correlated and negatively predict anxiety in children. Mol. Psychiatr..

[56] Kagerbauer S., Martin J., Schuster T., Blobner M., Kochs E., Landgraf R. (2013). Plasma oxytocin and vasopressin do not predict neuropeptide concentrations in human cerebrospinal fluid. J. Neuroendocrinol..

[57] White‐Traut R., Watanabe K., Pournajafi‐Nazarloo H., Schwertz D., Bell A., Carter C.S. (2009). Detection of salivary oxytocin levels in lactating women. Dev. Psychobiol.: J. Int. Soc. Develop. Psychobiol..

[58] Feldman R., Gordon I., Zagoory‐Sharon O. (2011). Maternal and paternal plasma, salivary, and urinary oxytocin and parent–infant synchrony: considering stress and affiliation components of human bonding. Dev. Sci..

[59] Bello D., White-Traut R., Schwertz D., Pournajafi-Nazarloo H., Carter C.S. (2008). An exploratory study of neurohormonal responses of healthy men to massage. J. Alternative Compl. Med..

