# Supplementary material for: Longitudinal tracking of human plasma oxytocin suggests complex responses to moral elevation
Source: Compr Psychoneuroendocrinol. 2021 Dec 22;9:100105. doi: 10.1016/j.cpnec.2021.100105 (PMC9216598; doi:10.1016/j.cpnec.2021.100105)
Supplement: Multimedia component 1 [file mmc1.docx]

**Supplementary File**

1. Selection of Stimuli

**Methods**

In a pilot study, individuals not studied in the blood-draw study were presented with three morally uplifting and one control videos selected from a larger pool of videos. The study was conducted online using Qualtrics survey software. Following the presentation, they were asked to score from 1 *(didn’t feel at all)* to 9 *(felt very strongly)* on several emotional responses to the video: moved, uplifted, amused, optimistic about humanity, happy, ‘warm’ feeling in chest, want to help others, want to become a better person, lump in throat, tears welling up, and interested in the video. High and low score responses to the two items “want to become a better person” and “want to help others” were used to select the stimulus and the control videos, respectively.

**Results**

*Participants*

The online pilot study to test the stimulus videos included 158 participants of whom 130 were female and 28 were male. Participant ages were captured in 10-year ranges and were as follows: 19–24 (n=36), 25–34 (n=17), 35–44 (n=19), 45–54 (n=35), 55–64 (n=34), 65–74 (n=15), and 75+ (n=2) years of age.

*Self-Report Response*

The two moral elevation stimulus videos scored the highest (Thai Life: 7.32 ± 2.11, n=74; Oprah Winfrey: 6.28 ± 2.53, n=76) while two of the control videos scored the lowest (battery: 2.12 ± 2.06; stool: 2.10 ± 2.10) on the two criteria (“touched/inspired” and “happy/joyful”). An origami construction video was added *post hoc* to permit time-matching with the moral elevation stimulus presentation but was not tested in this study.

1. Group-average Plasma Oxytocin


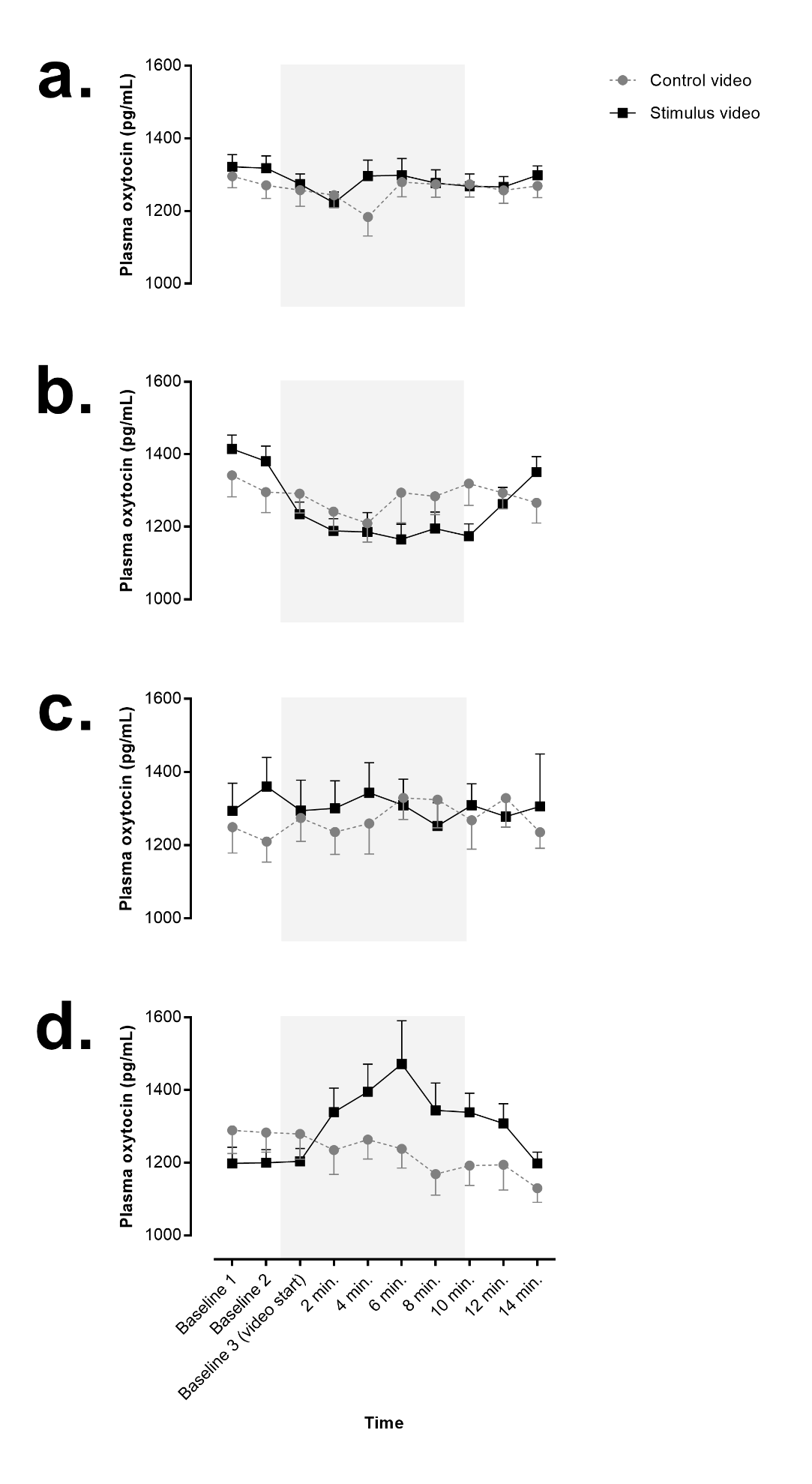

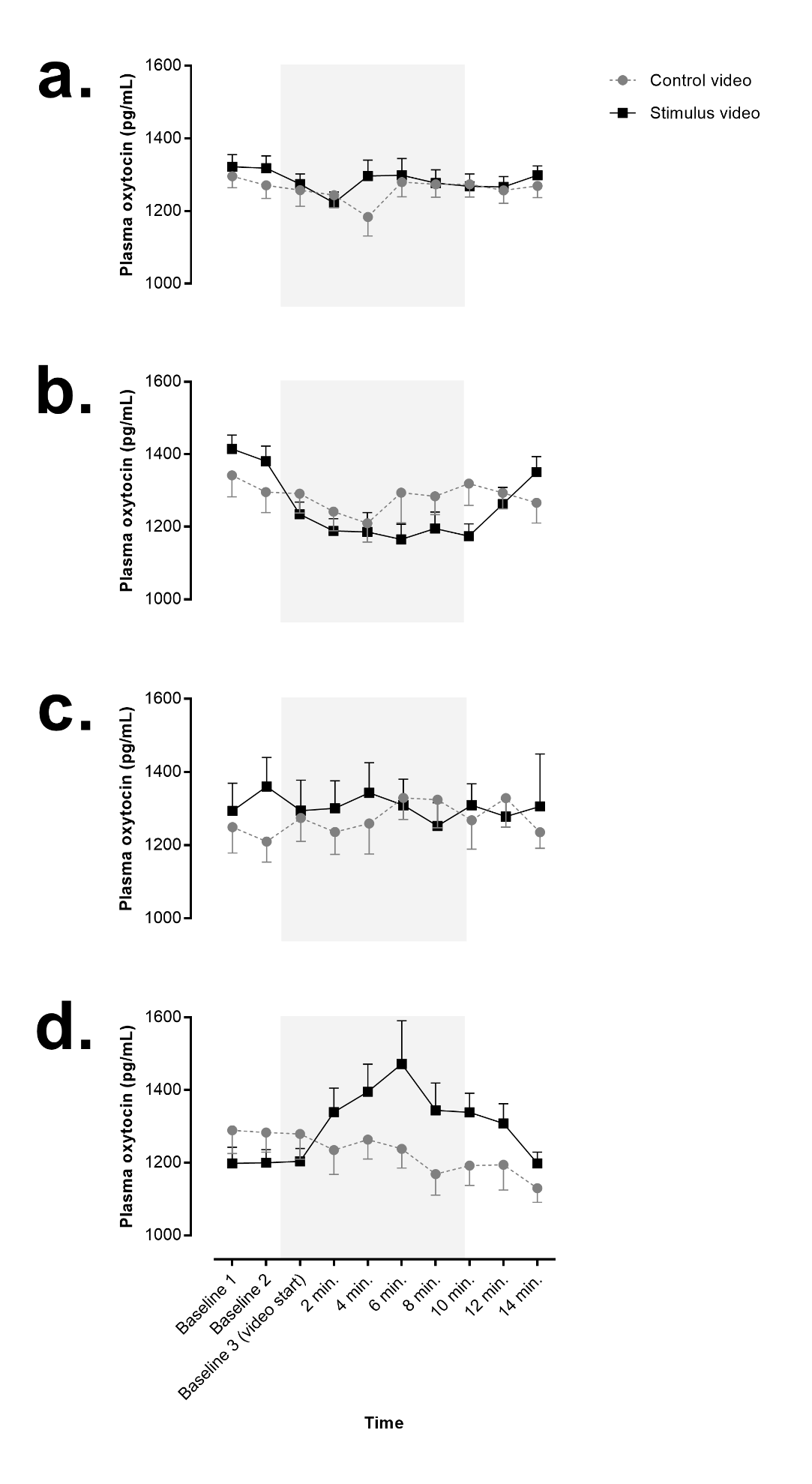


Figure 1: Plots showing plasma group-average oxytocin concentrations for each session. The COV Control condition is indicated by broken lines. The MEV Stimulus condition is indicated by solid lines.

1. Individual Plots of Plasma Oxytocin

Figure 2: Plots showing plasma oxytocin concentration for each individual. The COV Control condition is indicated by broken lines. The MEV Stimulus condition is indicated by solid lines.

1.
